# A Novel Nutraceutical Supplement Lowers Postprandial Glucose and Insulin Levels upon a Carbohydrate-Rich Meal or Sucrose Drink Intake in Healthy Individuals—A Randomized, Placebo-Controlled, Crossover Feeding Study

**DOI:** 10.3390/nu16142237

**Published:** 2024-07-11

**Authors:** Giriprasad Venugopal, Rishikesh Dash, Siwani Agrawal, Sayantan Ray, Prasanta Kumar Sahoo, Balamurugan Ramadass

**Affiliations:** 1 Center of Excellence for Clinical Microbiome Research, All India Institute of Medical Sciences Bhubaneswar, Bhubaneswar 751019, India; giriprsd36@gmail.com (G.V.); rishikeshdash50@gmail.com (R.D.); 2 Department of Biochemistry, All India Institute of Medical Sciences, Bhubaneswar 751019, India; snehisiwani@gmail.com; 3 Department of Endocrinology, All India Institute of Medical Sciences, Bhubaneswar 751019, India; endocrin_sayantan@aiimsbhubaneswar.edu.in; 4 Department of Ayurveda (AYUSH), All India Institute of Medical Sciences, Bhubaneswar 751019, India; ayush_prasanta@aiimsbhubaneswar.edu.in

**Keywords:** glucose lowering, glucose tolerance, hyperglycemia, carbohydrate-rich meal, sucrose drink, GLUBLOC^TM^

## Abstract

Background: Alkaloid- and polyphenol-rich white mulberry leaf and apple peel extracts have been shown to have potential glucose-lowering effects, benefitting the control of postprandial blood glucose levels. This study aimed to determine the effect of the combination of *Malus domestica* peel and *Morus alba* leaf extracts (GLUBLOC^TM^) on postprandial blood glucose and insulin-lowering effects in healthy adults after a carbohydrate-rich meal or sucrose drink intake. Methods: This study was designed as a randomized, crossover, single-blinded clinical trial. Out of 116 healthy participants, 85 subjects (aged 18–60 years) completed the day 1 and 5 crossover study. On day 1, subjects were supplemented with a placebo or GLUBLOC^TM^ tablet 10 min before the carbohydrate-rich meal (300 g of tomato rice) or sucrose drink intake (75 g of sucrose dissolved in 300 mL water). On day 5, the treatments were crossed over, and the same diet was followed. Postprandial blood glucose and insulin levels were measured on days 1 and 5 (baseline 0, post-meal 30, 60, 90, and 120 min). Differences in iAUC, Cmax, and Tmax were determined between the placebo and GLUBLOC^TM^-treated cohorts. Results: Significant changes in total iAUC (0–120 min), Cmax, and Tmax of postprandial blood glucose and insulin levels were noticed upon GLUBLOC^TM^ supplementation. The percentage reduction in the iAUC of blood glucose levels was 49.78% (iAUC_0–60min_) and 43.36% (iAUC_0–120min_), respectively, compared with the placebo in the sucrose drink intake study. Similarly, there was a 41.13% (iAUC_0–60min_) and 20.26% (iAUC_0–120min_) glucose-lowering effect compared with the placebo in the carbohydrate-rich meal intake study. Conclusions: Premeal supplementation with GLUBLOC^TM^ significantly reduced the postprandial surge in blood glucose and insulin levels after a carbohydrate-rich meal or sucrose drink intake over 120 min in healthy individuals. This study proves that GLUBLOC^TM^ can manage steady postprandial blood glucose levels.

## 1. Introduction

India is one of the largest rice and table sugar consumers compared with the rest of the world [1]. In 2021, the International Diabetes Federation (IDF) estimated that approximately 74.2 million adults in India aged 20–79 years had diabetes and that India was the second-largest diabetic population in the world after China [2,3]. The prevalence of diabetes in India has been steadily rising because of factors such as changes in lifestyle, urbanization, and an aging population. Individual blood sugar targets vary depending on factors like age, overall health index, and the type of diabetes (type 1 or type 2) [4,5]. 

Recent studies have revealed a correlation between lower levels of postprandial blood glucose (PPG) and insulin (PPI) and a decreased likelihood of developing diabetes and cardiovascular diseases [6,7]. Further research has focused on identifying specific food components and dietary compositions that may effectively and quickly lower PPG and PPI at a reasonable cost.

One way to approach this problem is to find “functional” ingredients that effectively decrease postprandial glucose and insulin levels when consumed with foods high in glycemic carbohydrates. These ingredients may have a pre-absorptive impact by acting as natural inhibitors of enzymes (such as α-amylase and α-glucosidase) or transporters (such as sodium–glucose linked cotransporter 1 [SGLT1] and glucose transporter 2 [GLUT2]) involved in carbohydrate digestion and absorption. These physiological mechanisms have been suggested as potential targets for interventions that regulate the rate at which glucose is released or absorbed from the foods consumed [8,9,10,11]. Various plant extracts or combinations have shown potential in vitro effects against these targets, and, in some cases, there is clinical evidence to support their ability to regulate blood sugar levels [12,13]. Many of these may be available as supplements or used in traditional medicine.

In recent years, mulberry leaf extract has gained attention for its potential role in postprandial glucose and insulin management. White mulberry leaf extract contains compounds including 1-deoxynojirimycin (DNJ), which is described to have an alpha-glucosidase inhibitor activity and thus might inhibit the conversion of complex carbohydrates to simple sugars, thereby limiting the absorption of sugars in the digestive tract [14,15,16]. Studies suggest that mulberry leaf extract improves insulin sensitivity. Insulin sensitivity refers to the body’s ability to use insulin effectively to transport glucose from the bloodstream into cells for energy. Improved insulin sensitivity helps to manage glucose levels [17,18,19,20]. Apple peel extract supports slowing down the digestion and absorption of carbohydrates, leading to a more gradual rise in blood sugar levels after a meal. This effect is partly attributed to the polyphenols and fiber in apple peel and partly to its phlorizin content, a competitive inhibitor of SGLT1 and SGLT2 [21,22,23,24,25]. 

The aim of this randomized, crossover study was to determine the positive impact of a novel combination of white mulberry leaf and green apple peel extracts (GLUBLOC^TM^) in lowering postprandial blood glucose and insulin levels after a carbohydrate-rich meal or sucrose drink intake in healthy individuals. 

## 2. Methods

Investigatory product—Each GLUBLOC^TM^ tablet contains a 500 mg proprietary blend of aqueous extracts of *Morus alba* L and *Malus domestica* rind, standardized to 5% 1,5-dideoxy-1,5-imino-D-sorbitol hydrochloride (1-DNJ) with ≥10% polyphenols (Supplementary Table S1). GLUBLOC^TM^ tablets were generously provided by My PuraVida Wellness Pvt Ltd., Hyderabad, Telangana, India. The product is manufactured following strict current Good Manufacturing practices.

Study setting—This study was conducted at the tertiary care hospital, AIIMS Bhubaneswar, Odisha, India. This study was designed as a randomized, crossover, single-blind clinical trial. Healthy subjects were recruited among healthcare workers and students at AIIMS Bhubaneswar. This study was approved by the institutional ethics committee of AIIMS Bhubaneshwar with the approval number T/EMF/Biochem/22/109. This trial was registered in the Clinical Trials Registry India (http://ctri.nic.in, accessed on 15 May 2023), with the reference number CTRI/2023/05/052654.

**Figure 1:**
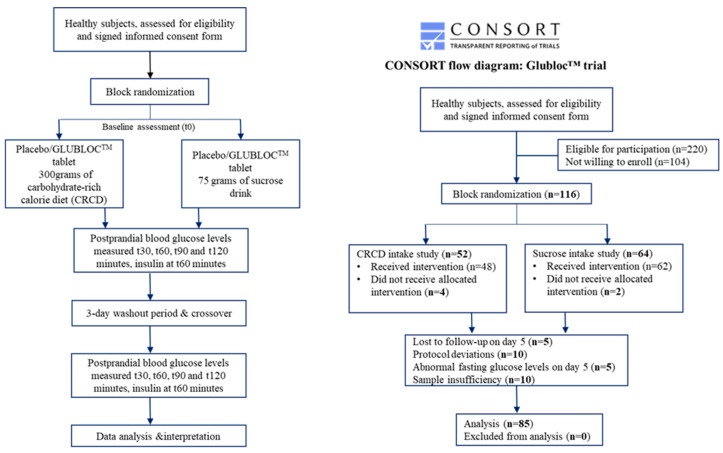
CONSORT flow diagram showing the study setup, enrollment, allocation, and analysis.

Participant Recruitment—In total, 368 healthcare workers and students were screened for this study. Following the inclusion and exclusion criteria, 116 participants were enrolled. The enrolled population in this clinical trial belongs to Odisha’s low to middle socioeconomic status group [26]. They eat a rice-based diet with a high carbohydrate and low fiber intake that describes standard dietary habits. After obtaining informed consent, subject demographics and medical history were collected using a Clinical Record Form. Blood parameters such as ALT, AST, and creatinine (baseline) were measured for all the study participants. Of the 116 participants, 85 completed the final assessments and were included for analysis (Figure 1).

The inclusion criteria included the following: 1. Aged between 18 and 60 years. 2. BMI between 18.5 and 22.9 kg/m^2^. 3. Fasting blood glucose between 3.9 and 5.5 mmol/L or 70 mg/dL to 99 mg/dL.

The exclusion criteria included the following: (1) Women who were pregnant or lactating. (2) Any known food allergies. (3) Subjects with a bleeding disorder. (4) Pre-existing medical conditions or taking medication that are known to affect glucose regulation and influence digestion and nutrient absorption. (5) History of diabetes mellitus (type 1/2) or use of antihyperglycemic drugs or insulin to treat diabetes or related conditions. (6) Use of steroids, protease inhibitors, or antipsychotic medicines, as these drugs are known to impact glucose metabolism and body fat distribution. (7) Refused consent.

Blinding and Randomization—All the study participants were blinded. The participants were allocated and provided with unique study numbers according to the random number generated by an online-based random numbering tool. The allocated participant number was used to identify the participants and their corresponding intervention sequence. Two products were tested in this study as follows: placebo (tablet containing 500 mg of microcrystalline cellulose with 150 mg excipients) and the GLUBLOC™ tablet containing a 500 mg proprietary blend of Malus domestica peel and Morus alba leaf extract with 150 mg excipients (commercially available as MODERATE™, by My PuraVida Wellness Pvt Ltd., Hyderabad, India). The test and placebo tablets were given and asked to be consumed 10 min before the carbohydrate-rich meal (300 g of cooked tomato rice, equal to 400 calories) or sucrose drink (75 g of sucrose dissolved in 300 mL of water, equal to 300 calories) intake.

The placebo and test products were allocated to participants using a randomized method. All study subjects received the placebo or test products in random order on day 1, along with the carbohydrate-rich meal or sucrose drink, followed by the 3-day washout period, and follow-up intervention supplements were crossed over for day 5 (Figure 1).

Study methodology—On day 1, subjects were randomized to placebo and treatment arms following an overnight fast of at least 10 h. A butterfly needle was used for a one-time venipuncture, and blood was withdrawn using a disposable syringe at given time intervals (baseline and postprandial). All the study subjects were supplemented with either GLUBLOC™ or placebo tablets 10 min before the carbohydrate-rich meal or sucrose drink intake. Out of the 116 participants, 52 and 64 subjects were fed a carbohydrate-rich meal and a sucrose drink, respectively. The subjects were requested to consume the meal or drink within 15 min or less. However, post-study, there were no dietary restrictions for the rest of the day. The baseline 5 mL of blood and post-intervention samples were collected at 30, 60, 90, and 120 min. After the day 1 assessment, three days of washout were observed. The participants were advised to maintain their usual diet and lifestyle during this phase. On day 5, the crossover was performed using the same meal/drink in the respective participants. Among the 116 subjects, 31 subjects were excluded from this study because of the following reasons: (1) abnormal baseline blood glucose levels on day 5, (2) unwillingness to give consent on day 5, (3) insufficient blood for the insulin analysis, and (4) deviations during the conduct of this study (Figure 1).

Sample processing—Blood samples were collected in K3- and EDTA-coated tubes for plasma isolation and Vaku-8 Vacuum Blood Collection Tube Gel + BCA–Gold tubes for serum isolation. Samples were centrifuged at 4000 RPM for 10 min (Weswox WT-24 brushless non-refrigerated centrifuge), and serum and plasma were isolated from the samples and stored at −20 °C in a deep freezer for glucose and insulin analysis. Blood plasma was used to measure blood glucose levels, while blood serum was used to measure insulin levels and liver and kidney function markers (ALT, AST, and creatinine). Blood glucose, ALT, AST, and creatinine were measured using a clinical chemistry analyzer (VITROS^®^ 5600 Integrated System, Raritan, NJ, USA). Insulin levels were measured as per the manufacturer’s instructions; 50 µL of the serum sample was tested for insulin using the Human Insulin ELISA kit (BIOGENEIX INC. PVT. Ltd., Lucknow, India), based on the OD. The results were expressed in micro units (µU) per milliliter.

### Sample Size

A total of 100 patients were calculated for this two-treatment parallel-design study based on 20% dropout. The probability that this study detected a treatment difference at a one-sided 0.05 significance level was 90 percent. This was based on the assumption that the standard deviation of the response variable was 90.

## 3. Statistical Analysis

Demographic characteristics were calculated as the mean ± SEM (standard error mean). The *t*-test test was used to find statistical significance for continuous variables, while the chi-square test was used for categorical variables. The positive incremental area under the curve (iAUC) was calculated (0–30 min, 0–60 min, 0–90 min, and 0–120 min) for both glucose and insulin levels separately for the carbohydrate-rich meal and sucrose drink intake studies [27,28]. Total iAUC changes in blood glucose and insulin were calculated for both GLUBLOC™ and the placebo geometrically by applying the trapezoid rule. Cmax (maximum concentration) and Tmax (maximum time taken to reach peak concentration) were calculated by directly taking glucose and insulin values. The percentage change in Cmax and Tmax was calculated for the glucose and insulin levels. An ANOVA/*t*-test was used to find the significant differences, followed by Tukey’s HSD post hoc test to find the mean differences, and false discovery rate (FDR)-corrected *p*-values were calculated using the Benjamin Hochberg method. A significance level of *p* < 0.05 was considered. All statistical analyses were performed using R Version 4.3.1.

## 4. Results

A total of 116 subjects were recruited for this study. Out of the 116 participants, 85 subjects completed this study with GLUBLOC^TM^ or placebo with two different carbohydrate meals on days 1 and 5, and their data were used for further statistical analysis (Figure 1). Table 1 summarizes the demographic and clinical characteristics of the study participants for two different carbohydrate diets on days 1 and 5. No adverse effects, such as bloating, indigestion, diarrhea, or other side effects with placebo or GLUBLOC™ intake were reported during this study.

**Figure 2:**
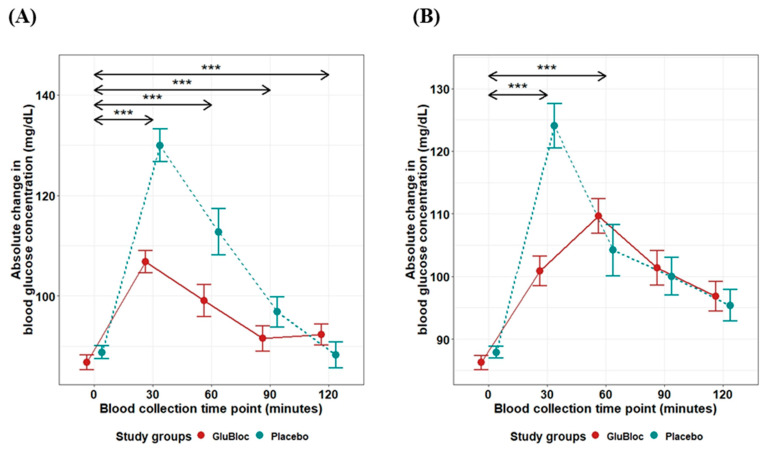
Glucose iAUC (mg/dL) over 2 h for GLUBLOC™ and placebo. (**A**) Sucrose drink intake values are the mean for 45 participants with SEM and (**B**) CRCD intake values are the mean for 40 participants with SEM. *p* < 0.05 is considered significant. SEM; standard error mean. (*** *p* < 0.0001).

The positive iAUC values (0–30, 0–60, 0–90, and 0–120 min) for postprandial glucose and insulin with the placebo and GLUBLOC™ treatments are shown in Table 2. Regarding the postprandial blood glucose levels in the sucrose drink arm (Figure 2A), when compared with the placebo, statistically significant differences in iAUC_0–60min_ and iAUC_0–120min_ were observed in the GLUBLOC™-treated group, with mean differences (95% CI) of ΔiAUC_0–60min_ 809.33 (491.53, 1127.12) mg/dL × min, *p* < 0.0001, and ΔiAUC_0–120min_ 1076.85 (484.38,1669.32) mg/dL × min, *p* < 0.001. In the carbohydrate-rich meal intake arm (Figure 2B), when compared with the placebo, a statistically significant difference in iAUC_0–60min_ was observed in the GLUBLOC™-treated group, with a mean difference (95% CI) of ΔiAUC_0–60min_ 560.86 (232.41, 889.32) mg/dL × min. However, no significant difference was found for iAUC changes from 0 to 120 min.

**Figure 3:**
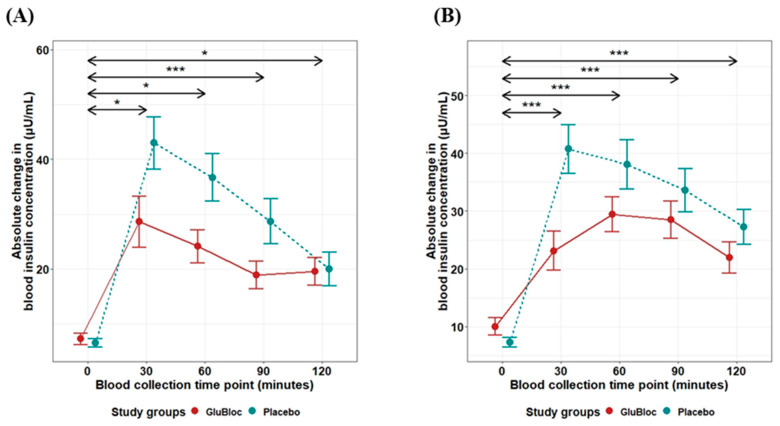
Insulin iAUC (µU/mL × min) over 2 h for GLUBLOC™ and placebo. (**A**) Sucrose drink intake values are the mean for 45 participants with SEM and (**B**) CRCD intake values are the mean for 40 participants with SEM. *p* < 0.05 is considered significant. SEM; standard error mean. (* *p* < 0.01, *** *p* < 0.0001).

The postprandial insulin levels in the sucrose drink arm are depicted in Figure 3A. When compared with the placebo, statistically significant differences in iAUC_0–60min_ and iAUC_0–120min_ were observed in the GLUBLOC™-treated group with mean differences (95% CI) of ΔiAUC_0–60min_ 647.37 (182.19, 1112.55) µU/mL × min, *p* < 0.01, and ΔiAUC_0–120min_ 1174.90 (347.28, 2002.52) µU/mL × min, *p* < 0.01. In the carbohydrate-rich meal intake arm (Figure 3B), when compared with the placebo, a statistically significant difference in iAUC_0–60min_ and iAUC_0–120min_ was observed in the GLUBLOC™-treated group, with mean differences (95% CI) of ΔiAUC_0–60min_ 764.79 (405.56, 1124.02) µU/mL × min, *p* < 0.0001, and ΔiAUC_0–120min_ 1247.31 (543.33, 1951.29) µU/mL × min, *p* < 0.001.

In the sucrose drink intake arm, the percentage reductions in postprandial iAUC glucose values in the GLUBLOC™ = treated group when compared with the placebo treatment were found to be 50.81% (0–30 min), 49.78% (0–60 min), 47.68% (0–90 min), and 43.36% (0–120 min). In line with the glucose values, the percentage reductions in postprandial insulin iAUC values in GLUBLOC™ treated group when compared with the placebo were found to be 40.9% (0–30 min), 41.82% (0–60 min), 42.92% (0–90 min) and 40.96% (0–120 min) (Table 2). In the carbohydrate-rich meal intake arm, the percentage reductions in postprandial iAUC glucose values in GLUBLOC™ treated group when compared with the placebo treatment were found to be 58.02% (0–30 min), 41.13% (0–60 min), 26.07% (0–90 min) and 20.26% (0–120 min). In line with the glucose values, the percentage reductions in postprandial insulin iAUC values in GLUBLOC™ treated group when compared with the placebo were found to be 59.82% (0–30 min), 52.19% (0–60 min), 44.62% (0–90 min) and 41.3% (0–120 min) (Table 2). 

**Figure 4:**
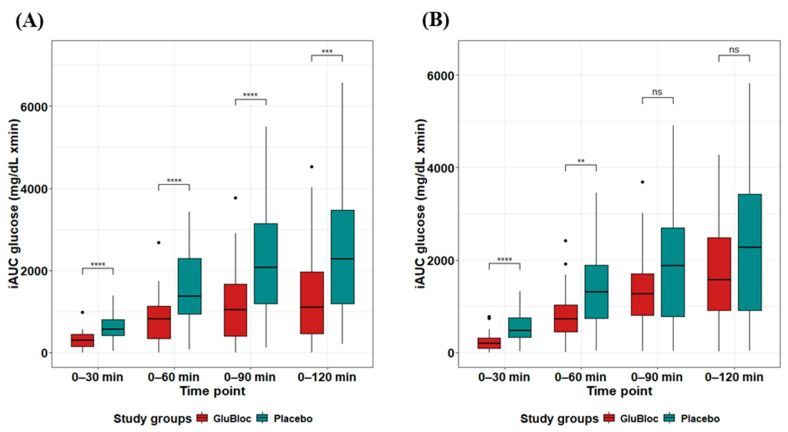
Glucose iAUC (mg/dL × min) values for 0–30, 0–60, 0–90, and 0–120 min. (**A**) Sucrose drink intake values are the mean for 45 participants with SEM and (**B**) CRCD intake values are the mean for 40 participants with SEM in GLUBLOC™ and the placebo. *p* < 0.05 is considered significant. SEM; standard error mean. (** *p* < 0.001, *** *p* < 0.0001, **** *p* < 0.00001, and ns: not significant).

Incremental area under the curve (iAUC) changes over 0–30, 0–60, 0–90, and 0–120 min for glucose levels between GLUBLOC™ and the placebo are shown in Figure 4A for the sucrose drink intake group and in Figure 4B for the carbohydrate-rich meal intake group. Statistically significant differences were identified between GLUBLOC™ and the placebo iAUC over 0–30 min, 0–60 min, 0–90 min, and 0–120 min in the sucrose drink intake group. Similarly, in the carbohydrate-rich meal intake group, statistically significant differences were identified between the GLUBLOC™ and placebo iAUC changes over 0–30 min and 0–60 min. However, no significant difference was identified for iAUC changes at 0–90 and 0–120 min (Table 2).

**Figure 5:**
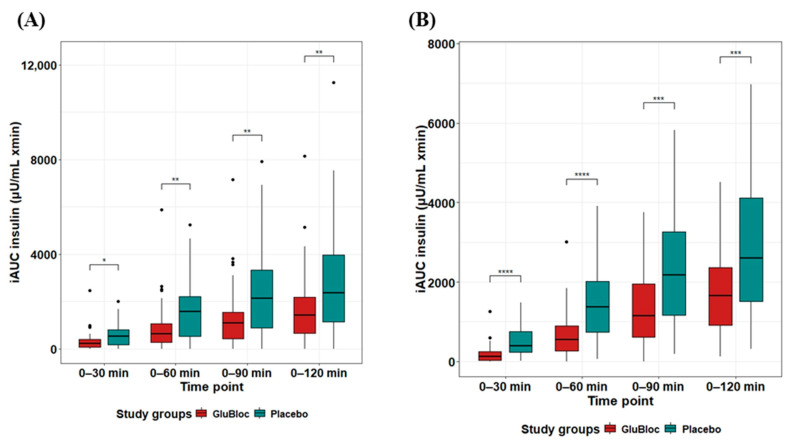
Insulin iAUC (µU/mL) values for 0–30, 0–60, 0–90, and 0–120 min. (**A**) Sucrose drink intake values are the mean for 45 participants with SE, and (**B**) CRCD intake values are the mean for 40 participants with SEM in GLUBLOC™ and the placebo. *p* < 0.05 is considered significant. SEM; standard error mean. (* *p* < 0.001, ** *p* < 0.01, *** *p* < 0.0001, **** *p* < 0.00001, and ns: not significant).

Similarly, iAUC changes over 0–30, 0–60, 0–90, and 0–120 min for insulin levels between GLUBLOC™ and the placebo were shown for the sucrose drink intake group (Figure 5A) and the carbohydrate-rich meal intake group (Figure 5B). There was a statistically significant difference in insulin levels between the GLUBLOC™ and placebo iAUC changes over 0–30 min, 0–60 min, 0–90 min, and 0–120 min in the sucrose drink intake group. In the carbohydrate-rich meal intake group, statistically significant differences between GLUBLOC™ and the placebo for iAUC changes over 0–30 min, 0–60 min, 0–90 min, and 0–120 min were identified (Table 2).

The significant changes in Cmax and Tmax calculations are shown in Table 3. When compared with the placebo treatment, premeal supplementation with GLUBLOC™ significantly reduced the Cmax of postprandial glucose and insulin in both the sucrose drink intake group by 22.07 and 15.26 and the carbohydrate-rich meal intake group by 12.75 and 10.95, respectively. GLUBLOC™ supplementation did not show significant changes in Tmax values of either postprandial glucose, 0.67, or insulin, −5.33, in the sucrose drink intake group when compared with the placebo treatment. Interestingly, GLUBLOC™ intake showed statistically significant changes in Tmax values of both glucose, 23.25, and insulin, −12, levels in the carbohydrate-rich meal intake group when compared with the placebo (Table 4).

## 5. Discussion

Traditionally, mulberry leaves and apple peel have a rich dietary importance globally and are consumed in several parts of Asia and Europe to regulate dietary blood glucose levels [29,30]. Mulberry leaves are rich in flavonoids, polyphenols, and alkaloids (fagomine and 1-DNJ), which have been shown to inhibit carbohydrate digestive enzymes, in particular, alpha-glucosidase, pancreatic α-amylase, and sucrase in the small intestine, which are responsible for the enzymatic hydrolyzation of polysaccharides, oligosaccharides, and disaccharides to monosaccharides [17,29,31]. Similarly, apple peel contains dietary polyphenols (quercetin, rutin, and phlorizin) known to not only inhibit carbohydrate-hydrolyzing enzymes and also block the transporters involved in glucose uptake (SGLT and GLUTs) at the brush border of the small intestine [12,21,22,23,24,25,32].

Even though several studies were conducted on white mulberry leaf preparations to understand their impact on postprandial blood glucose levels, most were performed with less statistical power or using smaller cohorts [33,34,35,36]. Apples are highly consumed and known for their beneficial impact in moderating blood glucose levels; however, their impact on postprandial blood glucose levels is poorly understood [22,24,25]. Research studies suggest that isoflavones improve insulin sensitivity, reduce inflammation, and enhance endothelial function. Specifically, mulberry leaves, rich in quercetin and kaempferol, exhibit potent antioxidant and anti-inflammatory properties, which help mitigate oxidative stress and improve metabolic health, potentially reducing the risk of diabetes and obesity. Similarly, apple peel contains flavonoids such as phloretin and epicatechin, which are known for their beneficial effects on lipid profiles, blood pressure, and vascular function [37,38]. Hence, we conducted this crossover study in 116 healthy participants, of which 85 subjects completed both day 1 and day 5 assessments for sucrose drink intake and carbohydrate-rich meal intake.

Moreover, GLUBLOC^TM^ (a novel blend of white mulberry leaf and apple peel extract), standardized to 5% 1-DNJ and with >10% polyphenols, showed superiority in inhibiting the carbohydrate-digesting enzymes α-amylase and α-glucosidase when compared with individual extracts of mulberry leaf and apple peel (Supplementary Figure S2) with IC50 values of 0.26 µg/mL and 0.28 µg/mL for α- amylase and α-glucosidase, respectively. The IC50 value of the standard drug acarbose against α-amylase is 0.42 µg/mL, and for α-glucosidase is 0.45 µg/mL (Supplementary Figure S1). Pancreatic amylase and α-glucosidase are particularly responsible for the breakdown of complex carbohydrates into simple sugars at the brush border of the intestine. The primary mechanism of Glubloc™ is its potential to temporarily inhibit the breakdown of carbohydrates, thereby limiting their availability for absorption [39,40]. 

We identified that GLUBLOC^TM^ intake reduced blood glucose iAUC by 49.78% and 43.36% for 0–60 min and 0–120 min, respectively, when compared with the placebo in the sucrose intake cohort. Similarly, GLUBLOC^TM^ supplementation reduced blood glucose iAUC by 41.13% and 20.26% for 0–60 min and 0–120 min, respectively, compared with the placebo in the carbohydrate-rich meal intake cohort. These findings indicate that GLUBLOC^TM^ limits glucose uptake from the small intestine. Moreover, we did not observe any side effects with GLUBLOC^TM^ administration, and no subjects dropped out of this study because of side effects or adverse events. This could be particularly beneficial for the population who tend to consume meals rich in carbohydrates and people with a tendency to develop prediabetes, diabetes, or weight gain because of metabolic dysregulation [41,42]. 

On sensing blood glucose, insulin is released from the pancreas to signal insulin-dependent glucose uptake by translocating GLUT-4 in the muscle cells, liver, and other major organs, further utilized by the mitochondria to generate ATP [43,44,45]. Hyperinsulinemia occurs when the organs become insulin-resistant and do not recognize the insulin response to glucose uptake [41,42,43,44]. GLUBLOC^TM^ intake reduced blood insulin levels by 41.82% and 40.96% for 0–60 min and 0–120 min, respectively, compared with the placebo in the sucrose intake cohort. 

Similarly, GLUBLOC^TM^ intake reduced insulin iAUC by 52.19% and 41.30% for 0–60 min and 0–120 min, respectively, compared with the placebo in the carbohydrate-rich meal intake cohort. These findings indicate that postprandial plasma insulin levels are significantly low because of less glucose availability upon GLUBLOC^TM^ intake. This is particularly useful for individuals with type 2 diabetes with insulin resistance, where hyperinsulinemia occurs because of a surge in postprandial blood sugar levels.

Refined carbohydrates with a high glycemic index (GI) raise blood glucose levels sharply, leading to high glycemic and insulinemic impact on the body [40,46]. Both rice and sucrose fall into the category of high-GI foods with a glycemic load (GL) equivalent to 56 for rice and 63 for table sugar [47]. We identified that GLUBLOC^TM^ intake before rice meals or sucrose drinks reduced the blood glucose Cmax of the respective foods and shifted Tmax to later time points compared with placebo intake (Table 3 and Table 4). These changes indicate that GLUBLOC^TM^ has the potential to shift the glycemic load of high-GI foods towards the low-GI category by delaying carbohydrate conversion and glucose absorption into the body.

The effects of GLUBLOC^TM^ may be enhanced in conjunction with dietary habits, physical activity, and the gut microbiome, potentially leading to better management of conditions like type 2 diabetes mellitus and improved long-term health outcomes [48]. GLUBLOC^TM^ supplementation led to a more gradual rise and decrease in post-meal sugar levels; these shifts may be beneficial when taken with foods or drinks with a high glycemic index. The Diabetes Prevention Program recommends lifestyle changes to prevent diabetes significantly in individuals with impaired glucose tolerance [33,46]. This study builds the body of evidence and supports the use of GLUBLOC^TM^ as part of a lifestyle change that may help manage postprandial blood glucose levels in healthy individuals with diabetes and prediabetes, especially when they look forward to consuming a high-carbohydrate or refined sugar-rich diet.

Diets rich in fiber, polyphenols, and other bioactive compounds promote beneficial microbial populations, which enhance insulin sensitivity and reduce inflammation. Conversely, high-fat, low-fiber diets can lead to dysbiosis, exacerbating insulin resistance and metabolic dysfunction. Furthermore, the gut microbiota can influence the metabolism and bioavailability of nutraceuticals and drugs, and vice versa, impacting their therapeutic effects. Gut microbiome studies are warranted on the implications of long-term consumption of GLUBLOC^TM^ as a dietary supplement for T2DM [49,50,51]. 

Limitations: The acute effects of a single dose of GLUBLOC^TM^ were only assessed in this study. Potential long-term benefits and side effects should be further explored. Additionally, this study only looked at the impact of premeal GLUBLOC^TM^ using a carbohydrate-rich meal and a sucrose drink in fasting healthy individuals without considering how it may interact with fats and proteins in a mixed diet. It is essential to understand the effects of GLUBLOC^TM^ on different types of carbohydrates, as they all contribute to the glycemic response and require different enzymes for digestion. It is worth noting that this study was conducted in participants with normal blood sugar levels, so caution should be exercised when applying the results to individuals with prediabetes or diabetes. Moreover, the possible effects of co-ingestion versus premeal ingestion of GLUBLOC^TM^ will be considered for future evaluations.

## 6. Conclusions

We demonstrated that GLUBLOC^TM^ (a proprietary blend of *Malus domestica* peel and *Morus alba* L. extracts) had a significant postprandial blood glucose-lowering effect and associated decrease in total insulin levels, as determined by iAUC. None of the participants reported any side effects, and no adverse events were recorded during this study. This study builds the body of evidence that supports the use of GLUBLOC^TM^ as part of a lifestyle change that may lead to maintaining normal postprandial blood glucose levels in healthy individuals as well as individuals with diabetes and prediabetes, especially when they look forward to consuming a high-carbohydrate and or a refined sugar-rich diet. 

## Figures and Tables

**Table 1:** Demographic and clinical characteristics (mean + SEM) of the study subjects.

Variable	Sucrose Drink Intake Group (*n* = 45)			CRCD Intake Group (*n* = 40)		
	Day 1 (*n* = 45)	Day 5 (*n* = 45)	*p*-Value	Day 1 (*n* = 40)	Day 5 (*n* = 40)	*p*-Value
Age (Yrs)	28.6 ± 0.96	__	NA	27.7 ± 1.14	__	NA
Gender M, F (%)	39, 6	__	NA	33, 7	__	NA
BMI (Kg/m^2^)	21.9 ± 0.21	__	NA	21.7 ± 0.26	__	NA
Fasting glucose (mg/dL)	89.6 ± 1.43	85.9 ± 1.3	0.22	85.9 ± 1.06	88.2 ± 1	0.22
Fasting insulin (µU/mL)	7.38 ± 1.09	6.31 ± 0.69	0.41	7.45 ± 0.73	9.79 ± 1.57	0.24

**Table 2:** iAUC changes (mean + SEM) for postprandial glucose and insulin.

Parameter		Sucrose Drink Intake Group (*n* = 45)			
		GLUBLOC™	Placebo	Percentage Change (%)	*p*-Value
Glucose mg/dL × min	0–30 min	304 ± 30.68	618 ± 44.99	50.81	<0.0001
	0–60 min	816.47 ± 86.01	1625.81 ± 134.82	49.78	<0.0001
	0–90 min	1161.10 ± 137.09	2219.18 ± 204.52	47.68	<0.0001
	0–120 min	1406.19 ± 178.25	2483.04 ± 238.98	43.36	<0.001
Insulin µU/mL × min	0–30 min	323.85 ± 61.76	547.95 ± 66.17	40.9	<0.05
	0–60 min	900.49 ± 152.7	1547.86 ± 177.41	41.82	<0.01
	0–90 min	1331.7 ± 197.48	2333.05 ± 27.62	42.92	<0.001
	0–120 min	1406.19 ± 178.25	2483.04 ± 238.98	40.96	<0.01
Parameter		CRCD Intake Group (*n* = 40)			
		GLUBLOC™	Placebo	Percentage Change (%)	*p*-Value
Glucose mg/dL × min	0–30 min	227.63 ± 30.12	542.25 ± 51.7	58.02	<0.0001
	0–60 min	802.76 ± 84.5	1363.62 ± 141.7	41.13	<0.001
	0–90 min	1395.47 ± 134.31	1887.52 ± 208.08	26.07	0.05
	0–120 min	1812.80 ± 173	2273.40 ± 252.84	20.26	0.13
Insulin µU/mL × min	0–30 min	201.73 ± 37.96	502.04 ± 56.38	59.82	<0.0001
	0–60 min	700.76 ± 98.24	1465.56 ± 151.35	52.19	<0.0001
	0–90 min	1286.82 ± 142.15	2323.62 ± 234.58	44.62	<0.001
	0–120 min	1693.02 ± 232.60	2867.92 ± 345.45	41.3	<0.001

**Table 3:** Cmax and Tmax of postprandial blood glucose and insulin.

Sucrose Drink Intake Group (*n* = 45)				CRCD Intake Group (*n* = 40)		
Cmax (mg/dL)				Cmax (mg/dL)		
Parameter	GLUBLOC^TM^ (*n* = 45)	Placebo (*n* = 45)	*p*-Value (FDR-Corrected)	GLUBLOC^TM^ (*n* = 40)	Placebo (*n* = 40)	*p*-Value (FDR-Corrected)
Glucose (mg/dL)	111.8 ± 2.3	133.87 ± 3.54	0.000001	114.38 ± 2.53	127.13 ± 3.46	0.00401
Insulin (µU/mL)	34.83 ± 4.56	50.09 ± 5.06	0.0276	40.51 ± 3.79	51.47 ± 4.22	0.0571
Tmax (min)				Tmax (min)		
Glucose (mg/dL)	41.33 ± 3.06	42 ± 2.76	0.872	65.25 ± 4.41	42 ± 3.69	0.000127
Insulin (µU/mL)	54.67 ± 4.60	49.33 ± 4.17	0.393	69.75 ± 4.35	57.75 ± 4.21	0.051

**Table 4:** Mean difference in Cmax and Tmax for glucose and insulin levels for GLUBLOC^TM^ vs. the placebo.

Diet	Parameter	Variable	Mean Difference	Lower 95 CI	Upper 95 CI	*p*-Value
Sucrose drink intake (*n* = 45)	Glucose	Cmax (mg/dL)	22.07	15.72	28.41	1.11 × 10^−8^
		Tmax (min)	0.67	−6.12	7.46	0.84
	Insulin	Cmax (µU/mL)	15.26	1.21	29.3	0.03
		Tmax (min)	−5.33	−18.55	7.88	0.42
CRCD intake (*n* = 40)	Glucose	Cmax (mg/dL)	12.75	6.46	19.04	0.0002
		Tmax (min)	23.25	11.06	35.44	0.0004
	Insulin	Cmax (µU/mL)	10.95	1.88	20.02	0.01
		Tmax (min)	−12	−24.06	0.06	0.05

## Data Availability

The data presented in this study are available on request from the corresponding author. The data are not publicly available due to intellectual property rights.

## Supplementary Materials

The following supporting information can be downloaded at: https://www.mdpi.com/article/10.3390/nu16142237/s1, Figure S1: Concentration-dependent enzymatic inhibition of α-glucosidase and α-amylase; Table S1: Quantitative analysis of Glubloc™ determined by GC-MS; File S1: Method.

## References

[1] Gulati S., Misra A. (2014). Sugar Intake, Obesity, and Diabetes in India. Nutrients.

[2] Sun H., Saeedi P., Karuranga S., Pinkepank M., Ogurtsova K., Duncan B.B., Stein C., Basit A., Chan J.C., Mbanya J.C. (2022). IDF Diabetes Atlas: Global, regional and country-level diabetes prevalence estimates for 2021 and projections for 2045. Diabetes Res. Clin. Pract..

[3] Atre S. (2019). The burden of diabetes in India. Lancet Glob. Health.

[4] Maiti S., Akhtar S., Upadhyay A.K., Mohanty S.K. (2023). Socioeconomic inequality in awareness, treatment and control of diabetes among adults in India: Evidence from National Family Health Survey of India (NFHS), 2019–2021. Sci. Rep..

[5] Ravikumar P., Bhansali A., Ravikiran M., Bhansali S., Walia R., Shanmugasundar G., Thakur J., Bhadada S.K., Dutta P. (2011). Prevalence and risk factors of diabetes in a community-based study in North India: The Chandigarh Urban Diabetes Study (CUDS). Diabetes Metab..

[6] Ruijgrok C., Blaak E.E., Egli L., Dussort P., Vinoy S., Rauh S.P., Beulens J.W., Robertson M.D., Alssema M. (2021). Reducing postprandial glucose in dietaryintervention studies and the magnitude of the effect on diabetes-related risk factors: Asystematic review and meta-analysis. Eur. J. Nutr..

[7] Rasmussen L., Poulsen C.W., Kampmann U., Smedegaard S.B., Ovesen P.G., Fuglsang J. (2020). Diet and Healthy Lifestyle in the Management of Gestational Diabetes Mellitus. Nutrients.

[8] Farrell T.L., Ellam S.L., Forrelli T., Williamson G. (2013). Attenuation of glucose transport across Caco-2 cell monolayers by a polyphenol-rich herbal extract: Interactions with SGLT1 and GLUT2 transporters. BioFactors.

[9] Kwon O., Eck P., Chen S., Corpe C.P., Lee J.-H., Kruhlak M., Levine M. (2007). Inhibition of the intestinal glucose transporter GLUT2 by flavonoids. FASEB J..

[10] Tattersall R. (1993). Alpha-glucosidase Inhibition as an Adjunct to the Treatment of Type 1 Diabetes. Diabet. Med..

[11] Wright E.M., Hirayama B.A., Loo D.F. (2007). Active sugar transport in health and disease. J. Intern. Med..

[12] Kashtoh H., Baek K.-H. (2022). Recent Updates on Phytoconstituent Alpha-Glucosidase Inhibitors: An Approach towards the Treatment of Type Two Diabetes. Plants.

[13] Bashkin A., Ghanim M., Abu-Farich B., Rayan M., Miari R., Srouji S., Rayan A., Falah M. (2021). Forty-One Plant Extracts Screened for Dual Antidiabetic and Antioxidant Functions: Evaluating the Types of Correlation between α-Amylase Inhibition and Free Radical Scavenging. Molecules.

[14] Takasu S., Parida I.S., Onose S., Ito J., Ikeda R., Yamagishi K., Higuchi O., Tanaka F., Kimura T., Miyazawa T. (2018). Evaluation of the antihyperglycemic effect and safety of microorganism 1-deoxynojirimycin. PLoS ONE.

[15] Asai A., Nakagawa K., Higuchi O., Kimura T., Kojima Y., Kariya J., Miyazawa T., Oikawa S. (2011). Effect of mulberry leaf extract with enriched 1-deoxynojirimycin content on postprandial glycemic control in subjects with impaired glucose metabolism: Mulberry DNJ and postprandial glycemia. J. Diabetes Investig..

[16] Kojima Y., Kimura T., Nakagawa K., Asai A., Hasumi K., Oikawa S., Miyazawa T. (2010). Effects of Mulberry Leaf Extract Rich in 1-Deoxynojirimycin on Blood Lipid Profiles in Humans. J. Clin. Biochem. Nutr..

[17] Chen S., Xi M., Gao F., Li M., Dong T., Geng Z., Liu C., Huang F., Wang J., Li X. (2023). Evaluation of mulberry leaves’ hypoglycemic properties and hypoglycemic mechanisms. Front. Pharmacol..

[18] Paudel P., Yu T., Seong S.H., Kuk E.B., Jung H.A., Choi J.S. (2018). Protein Tyrosine Phosphatase 1B Inhibition and Glucose Uptake Potentials of Mulberrofuran G, Albanol B, and Kuwanon G from Root Bark of *Morus alba* L. in Insulin-Resistant HepG2 Cells: An In Vitro and In Silico Study. IJMS.

[19] Niu S.-L., Tong Z.-F., Zhang Y., Liu T.-L., Tian C.-L., Zhang D.-X., Liu M.-C., Li B., Tian J.-L. (2020). Novel Protein Tyrosine Phosphatase 1B Inhibitor-Geranylated Flavonoid from Mulberry Leaves Ameliorates Insulin Resistance. J. Agric. Food Chem..

[20] Velasquez-Mieyer P.A., Cowan P.A., Arheart K.L., Buffington C.K., Spencer K.A., Connelly B.E., Cowan G.W., Lustig R.H. (2003). Suppression of insulin secretion is associated with weight loss and altered macronutrient intake and preference in a subset of obese adults. Int. J. Obes..

[21] Ci Z., Kikuchi K., Hatsuzawa A., Nakai A., Jiang C., Itadani A., Kojima M. (2018). Antioxidant Activity, and α-Glucosidase, α-Amylase and Lipase Inhibitory Activity of Polyphenols in Flesh, Peel, Core and Seed from Mini Apple. AJFST.

[22] Okada J., Yamada E., Okada K., Okada S., Yamada M. (2020). Comparing the efficacy of apple peels and a sodium-glucose cotransporter 2 inhibitor (ipragliflozin) on interstitial glucose levels: A pilot case study. Curr. Ther. Res..

[23] Niederberger K.E., Tennant D.R., Bellion P. (2020). Dietary intake of phloridzin from natural occurrence in foods. Br. J. Nutr..

[24] Blaschek W. (2017). Natural Products as Lead Compounds for Sodium Glucose Cotransporter (SGLT) Inhibitors. Planta Med..

[25] Yu C.H., Migicovsky Z., Song J., Rupasinghe H.V. (2023). (Poly)phenols of apples contribute to in vitro antidiabetic properties: Assessment of Canada’s Apple Biodiversity Collection. Plants People Planet.

[26] Venugopal G., Khan Z.H., Dash R., Tulsian V., Agrawal S., Rout S., Mahajan P., Ramadass B. (2023). Predictive association of gut microbiome and NLR in anemic low middle-income population of Odisha—A cross-sectional study. Front. Nutr..

[27] Lacey S. (2022). Incremental Area Under the Curve.

[28] Brouns F., Bjorck I., Frayn K.N., Gibbs A.L., Lang V., Slama G., Wolever T.M.S. (2005). Glycaemic index methodology. Nutr. Res. Rev..

[29] Jan B., Parveen R., Zahiruddin S., Khan M.U., Mohapatra S., Ahmad S. (2021). Nutritional constituents of mulberry and their potential applications in food and pharmaceuticals: A review. Saudi J. Biol. Sci..

[30] Hansawasdi C., Kawabata J. (2006). α-Glucosidase inhibitory effect of mulberry (*Morus alba*) leaves on Caco-2. Fitoterapia.

[31] Yatsunami K., Ichida M., Onodera S. (2007). The relationship between 1-deoxynojirimycin content and α-glucosidase inhibitory activity in leaves of 276 mulberry cultivars (*Morus* spp.) in Kyoto, Japan. J. Nat. Med..

[32] Khanam S., Mishra A., Shahid A., Pujari N.M. (2022). Therapeutic indication of Phloridzin: A new Gleam for metabolic disorders. Phytomed. Plus.

[33] Thondre P.S., Lightowler H., Ahlstrom L., Gallagher A. (2021). Mulberry leaf extract improves glycaemic response and insulaemic response to sucrose in healthy subjects: Results of a randomized, double blind, placebo-controlled study. Nutr. Metab..

[34] Kim J.Y., Ok H.M., Kim J., Park S.W., Kwon S.W., Kwon O. (2015). Mulberry Leaf Extract Improves Postprandial Glucose Response in Prediabetic Subjects: A Randomized, Double-Blind Placebo-Controlled Trial. J. Med. Food.

[35] Lown M., Fuller R., Lightowler H., Fraser A., Gallagher A., Stuart B., Byrne C., Lewith G. (2017). Mulberry-extract improves glucose tolerance and decreases insulin concentrations in normoglycaemic adults: Results of a randomised double-blind placebo-controlled study. PLoS ONE.

[36] Li M., Huang X., Ye H., Chen Y., Yu J., Yang J., Zhang X. (2016). Randomized, Double-Blinded, Double-Dummy, Active-Controlled, and Multiple-Dose Clinical Study Comparing the Efficacy and Safety of Mulberry Twig (Ramulus Mori, Sangzhi) Alkaloid Tablet and Acarbose in Individuals with Type 2 Diabetes Mellitus. Evid.-Based Complement. Altern. Med..

[37] Squadrito F., Marini H., Bitto A., Altavilla D., Polito F., Adamo E.B., D’Anna R., Arcoraci V., Burnett B.P., Minutoli L. (2013). Genistein in the metabolic syndrome: Results of a randomized clinical trial. J. Clin. Endocrinol. Metab..

[38] Ma L., Liu G., Ding M., Zong G., Hu F.B., Willett W.C., Rimm E.B., Manson J.E., Sun Q. (2020). Isoflavone Intake and the Risk of Coronary Heart Disease in US Men and Women: Results From 3 Prospective Cohort Studies. Circulation.

[39] Mudra M., Ercan-Fang N., Zhong L., Furne J., Levitt M. (2007). Influence of Mulberry Leaf Extract on the Blood Glucose and Breath Hydrogen Response to Ingestion of 75 g Sucrose by Type 2 Diabetic and Control Subjects. Diabetes Care.

[40] Kim G.-N., Kwon Y.-I., Jang H.-D. (2011). Mulberry Leaf Extract Reduces Postprandial Hyperglycemia with Few Side Effects by Inhibiting α-Glucosidase in Normal Rats. J. Med. Food.

[41] Bonsembiante L., Targher G., Maffeis C. (2021). Type 2 Diabetes and Dietary Carbohydrate Intake of Adolescents and Young Adults: What Is the Impact of Different Choices?. Nutrients.

[42] Hosseini F., Jayedi A., Khan T.A., Shab-Bidar S. (2022). Dietary carbohydrate and the risk of type 2 diabetes: An updated systematic review and dose–response meta-analysis of prospective cohort studies. Sci. Rep..

[43] Wilcox G. (2005). Insulin and insulin resistance. Clin. Biochem. Rev..

[44] Rahman S., Hossain K.S., Das S., Kundu S., Adegoke E.O., Rahman A., Hannan A., Uddin J., Pang M.-G. (2021). Role of Insulin in Health and Disease: An Update. IJMS.

[45] Leto D., Saltiel A.R. (2012). Regulation of glucose transport by insulin: Traffic control of GLUT4. Nat. Rev. Mol. Cell Biol..

[46] Diabetes Prevention Program Research Group (2009). 10-year follow-up of diabetes incidence and weight loss in the Diabetes Prevention Program Outcomes Study. Lancet.

[47] Vega-López S., Venn B.J., Slavin J.L. (2018). Relevance of the Glycemic Index and Glycemic Load for Body Weight, Diabetes, and Cardiovascular Disease. Nutrients.

[48] Shakappa D., Naik R., Sobhana P.P. (2022). Glycemic carbohydrates, glycemic index, and glycemic load of commonly consumed South Indian breakfast foods. J. Food Sci. Technol..

[49] Ramadass B., Rani B.S., Pugazhendhi S., John K.R., Ramakrishna B.S. (2017). Faecal microbiota of healthy adults in south India: Comparison of a tribal & a rural population. Indian. J. Med. Res..

[50] Antony M.A., Chowdhury A., Edem D., Raj R., Nain P., Joglekar M., Verma V., Kant R. (2023). Gut microbiome supplementation as therapy for metabolic syndrome. World J. Diabetes.

[51] Marini H.R. (2022). Mediterranean Diet and Soy Isoflavones for Integrated Management of the Menopausal Metabolic Syndrome. Nutrients.

