# Supplementary material for: A Novel Nutraceutical Supplement Lowers Postprandial Glucose and Insulin Levels upon a Carbohydrate-Rich Meal or Sucrose Drink Intake in Healthy Individuals—A Randomized, Placebo-Controlled, Crossover Feeding Study
Source: Nutrients. 2024 Jul 11;16(14):2237. doi: 10.3390/nu16142237 (PMC11280099; doi:10.3390/nu16142237)
Supplement: Supplementary file 1 [file nutrients-16-02237-s001.zip › nutrients-3076944-supplementary.pdf]

Supplementary data:

Methods:

1.1. GC–MS analysis

GC-MS analysis of Glubloc™ was conducted using a Hewlett-Packard 7890A gas chromatograph coupled to a mass detector 5975C quadrupole (Agilent, Palo Alto, CA, USA). The carrier gas, Helium, was allowed to flow at 1 mL/min. The column used was a TRB-1 (crosslinked methyl silicone) with dimensions of 30 m × 0.25 mm i.d. × 0.25 µm film thickness (Teknokroma). The oven temperature program was optimized, with the following conditions yielding the best results: the temperature was ramped at a rate of 15 °C/min from 100 °C to 200 °C, held at 200 °C for 15 minutes, then ramped at a rate of 15 °C/min to 300 °C and held at 300 °C for 10 minutes. The injector temperature was set at 300 °C, and injections were made in split mode with a split ratio 1:20. The mass spectrometer operated in electronic impact (EI) mode at 70 eV, scanning the 35-650 m/z range. The interface and source temperatures were set at 280 °C and 230 °C, respectively. Data acquisition was performed using HP ChemStation software from Hewlett-Packard. Linear retention indices (IT) were calculated based on the retention times of TMSO derivatives and suitable n-alkanes. Quantitative values were determined using the internal standard method. The limit of detection (LOD) was calculated as three times the signal-to-noise (S/N) ratio, while the limit of quantification (LOQ) was considered ten times this ratio(1,2).

1.2. Total Phenolic Content:

The Folin–Ciocalteu (FC) assay was performed on the Glubloc™ according to the method of Benelli et al. (2010). In each cuvette, 790 µL of MilliQ water, 150 µL of a 20% Na_2CO_3 solution, 50 µL of the FC reagent, and 10 µL of Glubloc™ solubilized in aqueous solution. The cuvettes were left in darkness for 2 hours, followed by spectrophotometric analysis, where the absorbance at 765 nm was measured for each sample. Gallic acid (0.25–2 g/L) served as a standard for calibration, and a linear regression line was constructed. The total phenolic content (TPC) was quantified as gallic acid equivalent (GAE) in milligrams per 100 g of the sample (3).

1.3. α-glucosidase and α-amylase enzyme inhibition assay

a) Alpha-glucosidase inhibitory activity of acarbose/GlublocTM was carried out according to the standard method(4). The reaction mixture containing 50 µL of phosphate buffer (100 mM, pH = 6.8), 10 µL alpha-glucosidase enzyme (1 U/mL), and 20 µL of varying concentrations (0.25 µg, 0.5 µg, 1 µg, 2.5 µg, 5 µg, 10 µg, 20 µg and 40 µg) of isolated compounds was pre-incubated at 37 °C for 15 min in 96-well plate. Next, 20 µL of p-NPG (5 mM) was added as a substrate and incubated further at 37 °C for 20 min. The reaction was stopped by adding 50 µL of sodium carbonate Na_2CO_3 (0.1 M). The absorbance of the released p-nitrophenol was measured at 405 nm using a Multiplate Reader (Epoch BioTek reader 2, Agilent Technologies, USA). Acarbose standards at different concentrations were included, and each experiment were performed in triplicates. Percentage inhibition is calculated using the formula:

   Inhibitory activity (%) = (1 − A/B) ×100

   Where A is the absorbance in the presence of the test substance, and B is the absorbance of the control.

b) The alpha-amylase inhibitory activity of acarbose/Glubloc™ was determined using the standard method(4). In a 96-well plate, 50 µL of phosphate buffer (100 mM, pH = 6.8), 10 µL of alpha-amylase enzyme (2 U/mL), and 20 µL of isolated chemical concentrations (0.25 µg, 0.5 µg, 1 µg,

2.5 µg, 5 µg, 10 µg, 20 µg, and 40 µg) were pre-incubated at 37 °C for 20 minutes. Next, 20 µL of 1% soluble starch (100 mM phosphate buffer pH 6.8) was added as a substrate and incubated at 37°C for 30 minutes. The reaction was stopped using 100 µL of color reagent (DNS) and by boiling for 10 minutes. The absorbance of the resultant combination was determined at 540 nm using a Multiplate Reader. Acarbose at varied concentrations served as a standard. Each experiment was carried out in triplicate. The data were presented as percentage inhibition, which was determined using formula.

Inhibitory activity (%) = $(1 - A/B) \times 100$

Where A is the absorbance in the presence of the test substance, and B is the absorbance of the control.

Results:

Supplementary Table S1. Quantitative analysis of Glubloc™ determined by GC-MS.

Compound	Mean Value (SD) (mg/g)
1,5-dideoxy-1,5-imino-D-sorbitol hydrochloride (1-DNJ)	51.28 (2.12)
Ribose	1.76 (0.29)
Fagomine	0.93 (0.01)
Pipecolic acid	trace*
Myo-inositol	9.15 (0.06)
Galactinols	2.74 (0.31)
Disaccharides	1.57 (0.14)
Phloridzin	4.32 (1.53)
Phloretin	1.85 (0.07)
Caffeic acid	0.07 (0.01)
p-Cumaric acid	trace*
Procyanidin B1+B3	0.16 (0.34)
Procyanidin B2	0.45 (0.12)
Epicatechin	1.54 (0.37)
Apigenin-7-O-glucoside	trace*
Neo-chlorogenic acid (3-CQA)	0.71 (0.02)
Chlorogenic acid (5-CQA)	8.32 (0.36)
Chlorogenic acid isomer (1-CQA)	1.33 (0.01)
Total Caffeoylquinic acids	12.64 (0.31)
Quercetin-3,7-O-β-glucopyranoside	0.74 (0.05)
Quercetin-3-O-β-glucopyranosyl-(1→6)-β-glucopyranoside	0.84 (0.04)
Quercetin-rutinoside isomer	0.23 (0.05)
Quercetin-rutinoside (rutin)	0.53 (0.01)
Quercetin-3-O-glucoside (isoquercitrin)	1.07 (0.12)
Quercetin-3-O-(6-malonyl)-β-glucopyranoside	1.74 (0.09)
Quercetin-3-O-6″-O-acetyl-β-glucopyranoside	0.89 (0.04)
Kaempferol-3,7-glucopyranoside	0.11 (0.11)
Kaempferol-3-O-rhamnoside	0.16 (0.07)
Kaempferol-3-O-6″-O-acetyl-β-glucopyranoside	0.39 (0.06)
Kaempferol-3-O-glucoside	9.17 (0.08)
Total polyphenol content	117.66 (0.24)
Values are expressed in mg/g (standard deviation (SD) of three repetitions.	

Supplementary Figure S1:

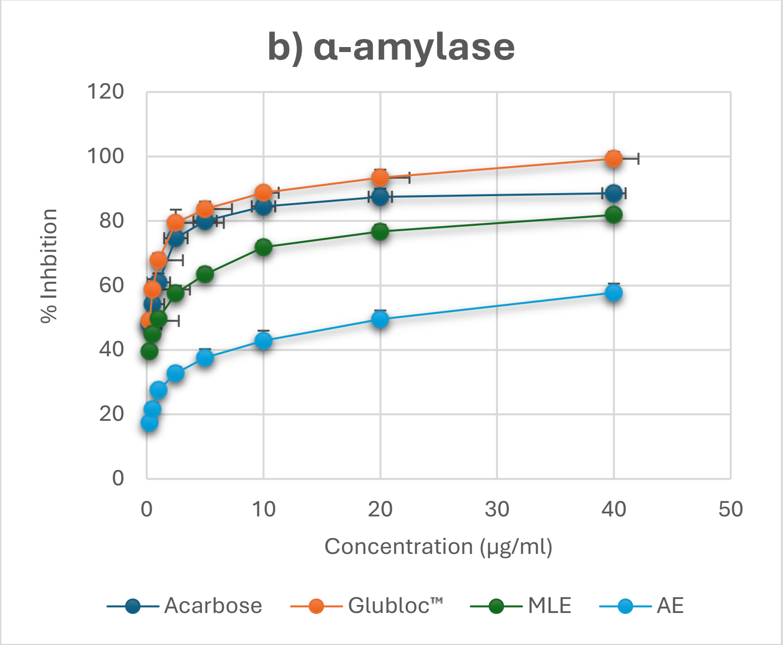

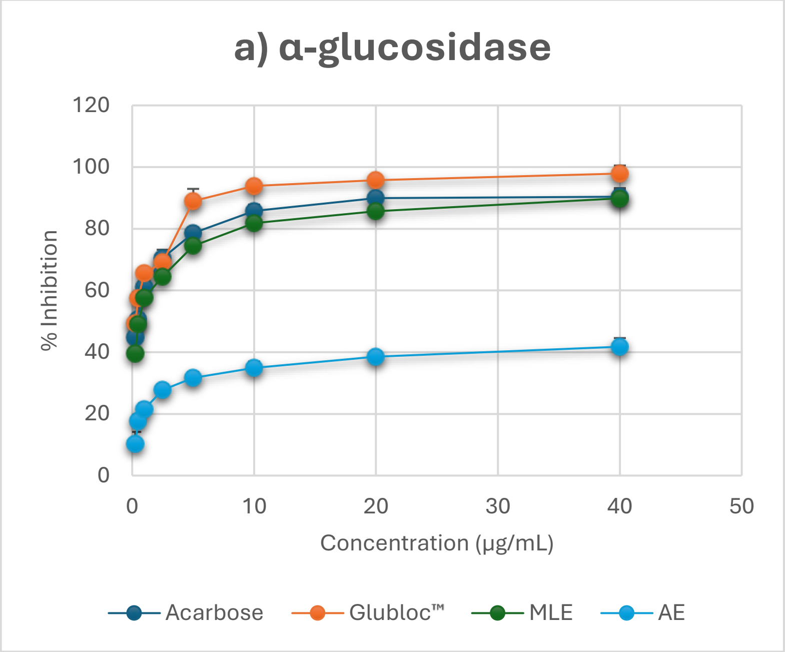

Supplementary Figure S1: a) Concentration-dependent enzymatic inhibition of α-glucosidase was carried out using Glubloc™ (green), Acarbose (red), Mulberry leaf extract- MLE (blue) and Apple extract- AE (orange). % inhibition values are represented as mean ± SEM; All the experiments are done in triplicates (n=3). b) Concentration-dependent enzymatic inhibition of α-amylase was carried out using Glubloc™ (green), Acarbose (red), MLE (blue), and AE (orange). % inhibition values are represented as mean ± SEM; All the experiments are done in triplicates (n=3).

Based on enzymatic inhibition assays, Glubloc™ exhibited IC50 values of 0.28 µg/mL and 0.26 µg/mL for α-glucosidase and α-amylase respectively. The IC50 value of standard drug acarbose against α-glucosidase is 0.45 µg/mL, and for α-amylase is 0.42 µg/mL. Pancreatic amylase and α-glucosidase are mainly responsible for the breakdown of complex carbohydrates into simple sugars at the brush border of the intestine. The primary mechanism of the Glubloc™ will be its potential to temporarily inhibit the breakdown of carbohydrates, thereby limiting its availability for absorption.

References:

1. Rodríguez-Sánchez S, Hernández-Hernández O, Ruiz-Matute AI, Sanz ML. A derivatization procedure for the simultaneous analysis of iminosugars and other low molecular weight carbohydrates by GC–MS in mulberry (Morus sp.). Food Chemistry. 2011 May;126(1):353–9.

2. Foley JP, Dorsey JG. Clarification of the limit of detection in chromatography. Chromatographia. 1984 Sep;18(9):503–11.

3. Benelli P, Riehl CAS, Smânia A, Smânia EFA, Ferreira SRS. Bioactive extracts of orange (Citrus sinensis L. Osbeck) pomace obtained by SFE and low pressure techniques: Mathematical modeling and extract composition. The Journal of Supercritical Fluids. 2010 Nov;55(1):132–41.

4. Hullatti K, Telagari M. In-vitro α-amylase and α-glucosidase inhibitory activity of Adiantum caudatum Linn. and Celosia argentea Linn. extracts and fractions. Indian J Pharmacol. 2015;47(4):425.
